# LONGITUDINAL MEASURES OF FRAILTY AND MORTALITY

**DOI:** 10.1093/geroni/igae098.1775

**Published:** 2024-12-31

**Authors:** Benjamin Seligman

**Affiliations:** University of California Los Angeles David Geffen School of Medicine, Los Angeles, California, United States

## Abstract

Assessment of frailty has become common in clinical settings to risk-stratify older adults. Understanding how to use repeated measurements answers important questions both for the clinical use of serial assessments and understanding frailty trajectories. Using the Health and Retirement Study, we calculated the most recent value, maximum, minimum, mean, standard deviation (SD), and slope of a deficit accumulation frailty index (FI) and the Fried frailty phenotype (FFP). We assessed the association of scaled values with mortality by Cox proportional hazards regression with adjustment for age, gender, and smoking status. We then used machine learning by LASSO regression to parsimoniously select variables associated with mortality. We found that the strongest association for the FI was its maximum value, with hazard ratio (HR) (95% CI) 1.67 (1.60-1.74). For FFP this was the mean value, HR 1.56 (1.44-1.70). For both FI and FFP, HRs of the most recent, maximum, and mean values were similar in magnitude, with estimates ranging from 1.62-1.67 and 1.50-1.56 respectively. In LASSO regression, the FI measures (HRs) retained were the maximum (1.38), most recent (1.18), and SD (1.01). For FFP, these were the mean (1.37), maximum (1.07), most recent (1.05). These findings show that maximum and most recent values of frailty tend to be most strongly associated with mortality. For clinicians, this means that the most recent assessment may be sufficient for many purposes. The associations of maximum frailty suggest that recovery may not lead to improved outcomes.

